# Association of Symptoms and Severity of Rift Valley Fever with Genetic Polymorphisms in Human Innate Immune Pathways

**DOI:** 10.1371/journal.pntd.0003584

**Published:** 2015-03-10

**Authors:** Amy G. Hise, Zachary Traylor, Noémi B. Hall, Laura J. Sutherland, Saidi Dahir, Megan E. Ermler, Samuel Muiruri, Eric M. Muchiri, James W. Kazura, A. Desirée LaBeaud, Charles H. King, Catherine M. Stein

**Affiliations:** 1 Center for Global Health and Diseases, Case Western Reserve University School of Medicine, Cleveland, Ohio, United States of America; 2 Department of Pathology, Case Western Reserve University School of Medicine, Cleveland, Ohio, United States of America; 3 Research Service, Louis Stokes Cleveland Department of Veterans Affairs Medical Center, Cleveland, Ohio, United States of America; 4 Department of Epidemiology and Biostatistics, Case Western Reserve University, Cleveland, Ohio, United States of America; 5 Division of Vector-Borne and Neglected Tropical Diseases, Ministry of Public Health and Sanitation, Nairobi, Kenya; 6 Division of Pediatric Infectious Diseases, UCSF Benioff Children's Hospital Oakland, Children's Hospital Oakland Research Institute, Oakland, California, United States of America; 7 Department of Pediatrics, Stanford University, Department of Pediatrics, Stanford, California, United States of America; Nagasaki University Institute of Tropical Medicine, JAPAN

## Abstract

**Background:**

Multiple recent outbreaks of Rift Valley Fever (RVF) in Africa, Madagascar, and the Arabian Peninsula have resulted in significant morbidity, mortality, and financial loss due to related livestock epizootics. Presentation of human RVF varies from mild febrile illness to meningoencephalitis, hemorrhagic diathesis, and/or ophthalmitis with residual retinal scarring, but the determinants for severe disease are not understood. The aim of the present study was to identify human genes associated with RVF clinical disease in a high-risk population in Northeastern Province, Kenya.

**Methodology/Principal Findings:**

We conducted a cross-sectional survey among residents (N = 1,080; 1–85 yrs) in 6 villages in the Sangailu Division of Ijara District. Participants completed questionnaires on past symptoms and exposures, physical exam, vision testing, and blood collection. Single nucleotide polymorphism (SNP) genotyping was performed on a subset of individuals who reported past clinical symptoms consistent with RVF and unrelated subjects. Four symptom clusters were defined: meningoencephalitis, hemorrhagic fever, eye disease, and RVF-not otherwise specified. SNPs in 46 viral sensing and response genes were investigated. Association was analyzed between SNP genotype, serology and RVF symptom clusters. The meningoencephalitis symptom phenotype cluster among seropositive patients was associated with polymorphisms in DDX58/RIG-I and TLR8. Having three or more RVF-related symptoms was significantly associated with polymorphisms in TICAM1/TRIF, MAVS, IFNAR1 and DDX58/RIG-I. SNPs significantly associated with eye disease included three different polymorphisms TLR8 and hemorrhagic fever symptoms associated with TLR3, TLR7, TLR8 and MyD88.

**Conclusions/Significance:**

Of the 46 SNPs tested, TLR3, TLR7, TLR8, MyD88, TRIF, MAVS, and RIG-I were repeatedly associated with severe symptomatology, suggesting that these genes may have a robust association with RVFV-associated clinical outcomes. Studies of these and related genetic polymorphisms are warranted to advance understanding of RVF pathogenesis.

## Author Summary

The underlying risk factors that lead to severe human Rift Valley Fever disease are unknown, but are likely multi-factorial. Host factors, such as innate immune genetic makeup, are likely important determinants of disease phenotype. This study investigated the association of 46 single nucleotide polymorphisms (SNPs) in genes encoding innate immune receptors, signaling pathways or mediators with RVF disease phenotype. Of the 46 SNPs tested, TLR3, TLR7, TLR8, MyD88, TRIF, MAVS, and RIG-I were repeatedly associated with severe RVF symptomatology, suggesting that these genes may have a robust association with RVFV-associated clinical outcomes.

## Introduction

Rift Valley fever virus (RVFV) is a negative strand RNA virus of the family *Bunyaviridae*. Episodic epidemics of Rift Valley Fever (RVF) present a significant natural threat to human health in many countries of Africa and the Middle East, causing retinitis, encephalitis and hemorrhagic fever [1,2]. Epizootics of RVFV also seriously affect livestock, including sheep, cattle, goats, buffalo, and camels, creating serious economic disruption and risk of famine [3]. Two of the largest RVF outbreaks have occurred in Kenya over the last decade, the first in 1997–98 [4], and another more recently in 2006–2007 [5]. Both epidemic human disease, (including hemorrhagic fever), and enzootic livestock disease, (including excess mortality and miscarriage), are most prevalent in semi-arid areas that experienced prolonged excess rainfall during El Nino-Southern Oscillation (ENSO) weather anomalies [6]. Given the recent US experience with West Nile Virus, we could expect that, after either accidental or intentional introduction, RVFV will have the potential to become a widespread multi-state or multinational problem in North America.

Our ongoing field studies aim to better define the epidemiology of RVF viral transmission at the local community level [7–11]. However, little is known about the pathogenesis of the variable disease progression observed between different RVFV-infected human subjects. In communities where 20–30% of persons are exposed, only about 1% of infections progress to severe liver dysfunction and hemorrhagic disease, and late onset encephalitis is rare, although 10–30% develop some form of anterior or retinal eye disease [12,13]. Currently there is no specific treatment for RVF.

It remains unclear why the majority of infected humans recover from RVFV infection after only a brief febrile illness. Evidence from experimental animal models suggests that early activation of innate immunity provides the greatest protection against lethal RVFV infection [14,15], and that differences in interferon-mediated response pathways [16–18] could be responsible for resistance to lethal infection [18–20]. Later-onset, adaptive immunity (with the production of neutralizing antibody responses) likely also plays a role in modulation of RVFV-infection associated disease. However, given the rapid time course of lethal disease progression, with most major symptoms developing within the first week of illness [21], a study of the variability in innate immune responses appears to be the logical first step to elucidate inter-subject variation in disease progression.

Several classes of innate receptors are important in host anti-viral defense, including membrane bound Toll-like Receptors (TLRs) [22], cytoplasmic DExD/H box RNA helicases such as retinoic acid-inducible gene-I (RIG-I) and melanoma differentiation-associated gene 5 (MDA5) [23–26] and NOD-like receptors (NLRs) including inflammasomes [27–32]. TLRs are innate receptors that recognize specific structures expressed by microorganisms. Surface TLR2 and −4 recognize viral proteins including hemagglutinin of measles virus [33] and components of HSV [34] and CMV [35] and RSV [36]. Endosomal TLRs act as receptors for nucleic acids, including TLR3 (dsRNA [37]); TLR7 and TLR8, (single-stranded RNA [38,39]); and TLR9, (unmethylated CpG DNA motifs [40]). There is strong evidence in support of a role for endosomal TLRs in the detection of viruses including Influenza virus and Vesicular Stomatitis virus (VSV) (TLR7) and HSV (TLR9 in certain cell types) [38,41–43]. Signaling by all TLRs originates from a conserved intracellular domain (Toll–IL-1–resistance; TIR), which mediates recruitment of members of a family of adaptor molecules. Recruitment of the common adaptor, myeloid differentiation factor 88 (MyD88) [44,45] leads to the interaction and activation of the IRAK family members [46] and the subsequent activation of TRAF6 [47] resulting in NF-κB activation. Activation of the Interferon Regulatory Factors (IRFs), important mediators of IFN gene transcription also occurs downstream of the TLRs. Thus, these pathways could potentially play an important role in modulating the severity of RVFV-associated disease in humans. A role for TLR3 was demonstrated in a murine model of another phlebovirus, Punta Toro virus [48]. Other reports have shown a protective role for poly I-C (ligand for TLR3 and MDA5) when used as a pretreatment prior to RVFV infection with virulent ZH501 strain [14,49]. In our previous study, TLRs did not play a predominant role in IFN production [50]; however, their role in human innate responses and RVF disease pathogenesis remains unclear.

Viral nucleic acid recognition can also occur via the RNA helicases RIG-I and MDA5 [23,24]. Both proteins are expressed in the cytoplasm and contain caspase recruitment domains (CARDs) as well as a C-terminal region harboring ATP-dependent RNA helicase activity [24]. RIG-I and MDA5 activate downstream signaling via the adaptor MAVS (mitochondrial anti-viral signaling [51], also called IPS1 [52,53], CARDif [54] or VISA [55]), which relays signals to downstream kinases to trigger IFN gene transcription. RIG-I is required for triggering anti-viral responses against several classes of RNA viruses including (*Flaviviridae, Paramyxoviridae, Orthomyxoviridae* and *Rhabdoviridae*) [52], whereas MDA5 is required for the response against picornaviruses like encephalomyocarditis virus (EMCV) [56,57]. RIG-I senses viral and synthetic RNA containing 5’-triphosphate caps whereas MDA5 detects synthetic poly(I-C) in vivo, although the nature of the viral ligand for MDA5 remains unclear [53,56,58]. Our previous work emphasized the importance of the RNA helicase adaptor MAVS for RVFV induced type I IFN production in primary immune cells as well as for protection against mortality and morbidity during mucosal challenge in mice [50]. We showed that type I IFN responses were mediated through RIG-I in mice and in vitro human cell systems, although MDA5 also played a role at the earliest time points of viral entry.

In the present study, we hypothesized that distinct RVFV-associated clinical syndromes are related to differences in early innate host responses to viral infection, and that variation in these host responses may be associated to differences in the makeup of innate immune response pathways. In this first genetic epidemiologic study of human RVF, we sought to examine the association between genes in innate immunity pathways and clinical phenotypes linked to acute RVFV infection. This manuscript describes the genetic associations we discovered among variants within host immunologic pathways that may influence susceptibility to RVFV-associated disease.

## Methods

### Ethics statement

The study protocol was approved by the University Hospitals Case Medical Center Institutional Review Board (IRB), Cleveland, Ohio (No. 11-09-01) and the Ethical Review Committee of the Kenya Medical Research Institute, Nairobi, Kenya (Non-SSC Protocol No. 195). Before participation, written informed consent was obtained from adult study subjects, and parents provided written informed consent for participating children; children over 7 years of age also provided individual assent.

### Study participants

**Fig 1:**
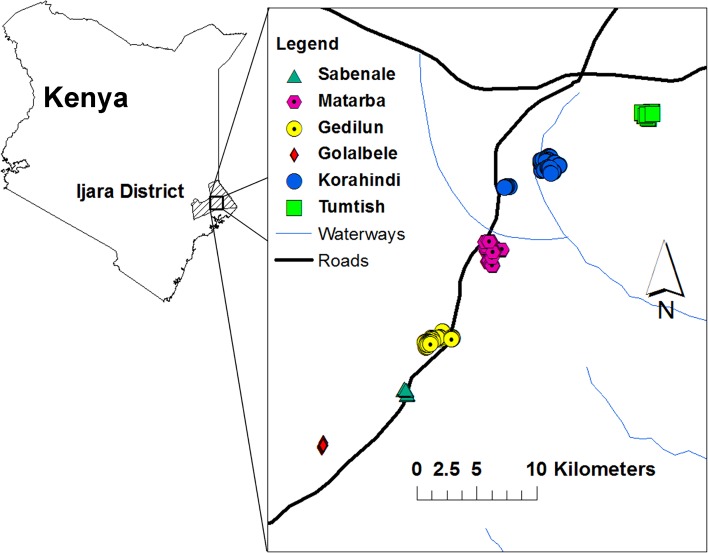
Map of the study area in Northeastern Province, Kenya. Shown are the locations of the Ijara District and, in the inset, the relative locations of the participating study villages in the Sangailu area of Kenya.

Study participants were recruited from six villages located in the Sangailu area of Ijara District, located in Northeastern Province, Kenya (Fig. 1). All local residents were eligible for participation, with the exception of those living in the area for less than 2 years, and children < 1 year of age, who were excluded. After an initial demographic census was performed, consented subjects were enrolled, surveyed via structured interview for potential RVFV exposure history and past symptoms suggestive of RVF, and examined by a nursing officer with particular attention to current visual acuity and eye disease, as previously described [10,59]. Whole blood was collected by phlebotomy (∼ 5 mL venous blood samples from persons > 5 years of age and 1 mL from children under the age of 5). Individual sera and associated blood clots were separated and stored frozen at −80°C.

The study sample was representative of the local mix of 99% Somali ethnicity, and < 1% Bantu, Indian, or other Asian. Out of 1134 household residents identified, 1128 completed survey questionnaires (parents served as proxies for young children), and 1110 completed a basic physical examination and vision check. A total of 1114 provided blood for anti-RVFV antibody screening, and 1082 provided full data from survey, exam and serology testing. This paper's genetic analysis focuses on 363 individuals from an RVF-endemic study area, Ijara Constituency of Garissa County. The study group included 117 individuals who were antibody-seropositive for RVFV and had DNA available for analysis, and 246 unrelated local control subjects from nearby households or villages. The study subjects (seropositive and seronegative) were sampled from 251 households in Sangailu Location (centered around coordinates 1 deg. 19 min S, 40 deg. 44 min E). In this area, residents per household ranged from 1 to 9 (median = 3), and for our study 1 to 4 persons were tested per household (average = 1.5 per household).

### Serology assay

To test for evidence of past RVFV infection, serum specimens were screened for the presence of anti-RVF IgG via ELISA [4,7]. Briefly, high protein binding plates (Corning) were coated with RVFV variant rMP-12 viral antigens prepared in Vero cell lysates, and blocked in 5% non-fat dairy milk. Serum samples were diluted 1:100 in PBS/5% milk solution and allowed to absorb for 1 h at 37°C. After washing, a HRP-conjugated secondary anti-human IgG antibody was applied, again in the milk solution at a 1:2000 dilution. Plates were incubated at 37°C for 1 h then developed using a TMB substrate (Thermo) and absorbance was read at 405nm. Each sample was run in duplicate, and OD values were normalized to background values from wells coated with uninfected Vero cell lysate and averaged. Samples were considered positive with OD values greater than the mean + 2 SD for pooled control sera obtained from unexposed North Americans.

### DNA extraction

Genomic DNA was isolated from frozen blood clots using a 96 well DNeasy Blood and Tissue kit (Qiagen) with some slight modifications. Approximately 500 μL of thawed blood clot material was homogenized in ALT lysis buffer using a mixer mill (Retsch). Proteinase K (Qiagen) was added to each tube and incubated at 56°C for 60 min with occasional agitation followed by pulse centrifuged at low speed to pellet debris. Supernatant was removed and DNA was eluted using spin columns according to the manufacturer’s recommendations. Total DNA was quantified using the Quant-iT PicoGreen dsDNA Assay Kit following the manufacturer’s recommendations (Life Technologies). The results were verified on random samples by spectrophotometry (NanoDrop 1000, Thermo Scientific). Low yielding samples were concentrated by re-precipitating the DNA in ethanol in the presence of 20mg/ml glycogen (Thermo Scientific) and re-suspended in TE buffer (Qiagen).

### SNP genotyping

This study focused on genes encoding molecules likely to be involved in early innate immune responses to RVFV including: IL6, IL6R, TLR3, TLR7, TLR8, TRIF (TICAM1), MyD88, RIG-I (DDX58), LGP-2 (DHX58), MAVS, IFNAR1, IFNB1, Mda5 (IFIH1), CCR5, DC-SIGN (CD209), and CFH. Single nucleotide polymorphisms (SNPs) within these genes were chosen if they were in the promoter region, the coding region of the gene, or in the untranslated regions (3’UTR or 5’UTR) of the gene, and having at least a 10% minor allele frequency in the Maasai (MKK) or Luhya (LWK) Kenyan HapMap populations [60–62]. The exception to this was CD209, where an intergenic SNP was chosen, as no putatively functional SNPs met the allele frequency criteria in HapMap. A total of 48 SNPs were genotyped using the Illumina VeraCode platform. Two SNPs failed quality control because of poor intensity; the remaining SNPs were all in Hardy-Weinberg equilibrium. Allelic frequencies are included in S1 Table.

### Clinical phenotype definitions

RVFV-related disease phenotypes were defined on the basis of subjects’ self-reported symptoms on the study intake questionnaires. The time period of interest for occurrence was any time during or after the 2006–2007 RVF epizootic in Northeastern Province. The nineteen questions included in the symptom review covered known RVF complications including isolated eye symptoms (eye pain, scleral injection, poor vision or blurry vision), central nervous system symptoms (photophobia, meningismus, vertigo/dizziness, reduced consciousness, confusion, coma, or seizures), symptoms of a hemorrhagic diathesis (bleeding gums, non-traumatic bruising, hematemesis, hematochezia), or non-focal systemic symptoms (fever, malaise, back ache, nausea, anorexia).

#### Symptom clusters

Subjects were initially categorized in heuristic system/severity groupings as having zero, one, or more than one (or two) of the symptoms in each of these system-related clusters (Table 1). For eye symptoms, subjects were classified as having zero, one (any_eye), or two or more eye symptoms (eye2). For meningoencephalitis symptoms, subjects were classified as having zero, one (ME-any), or 3 or more (ME3) central nervous system-related symptoms. For hemorrhagic fever symptoms, subjects were classified as having zero, one (HE-any) or three or more (HE3) symptoms. For non-focal symptoms, subjects were classified as zero, one (nonspec_any) or three or more (nonspec3) symptoms. Subjects were also categorized as having any RVF-related symptom (any) or any combination of three or more RVF-related symptoms (any3).

**Table 1:** Patient demographics and distribution of clinical phenotype clusters.

		N (%)
Positive serology	Yes	117 (32.3)
	No	245 (67.7)
Sex	Male	143 (33.8)
	Female	219 (67.2)
Meningoencephalitis symptoms—any (ME-any)	Yes	162 (44.6)
	No	201 (55.4)
Meningoencephalitis—3 or more symptoms (ME3)	Yes	30 (8.3)
	No	333 (91.7)
Hemorrhagic fever symptoms—any (HE-any)	Yes	34 (9.4)
	No	329 (74.1)
Hemorrhagic fever—3 or more symptoms (HE3)	Yes	4 (1.1)
	No	359 (98.9)
RVFV eye disease symptoms—any (any_eye)	Yes	140 (38.6)
	No	223 (50.2)
RVFV eye disease—2 or more symptoms (eye2)	Yes	107 (29.5)
	No	256 (57.7)
RVFV non-specific symptoms (nonspec_any)	Yes	318 (71.6)
	No	45 (12.4)
RVFV non-specific—3 or more symptoms (nonspec3)	Yes	159 (35.8)
	No	204 (56.2)
Any symptoms (any)	Yes	323 (89.0)
	No	40 (11.0)
Any 3 or more symptoms (any3)	Yes	207 (46.6)
	No	156 (43.0)
Symptom severity clusters:	Severe = 1	45 (38.5)
	Moderate = 2	44 (37.6)
	Mild = 3	28 (24)

#### Severity clusters

**Fig 2:**
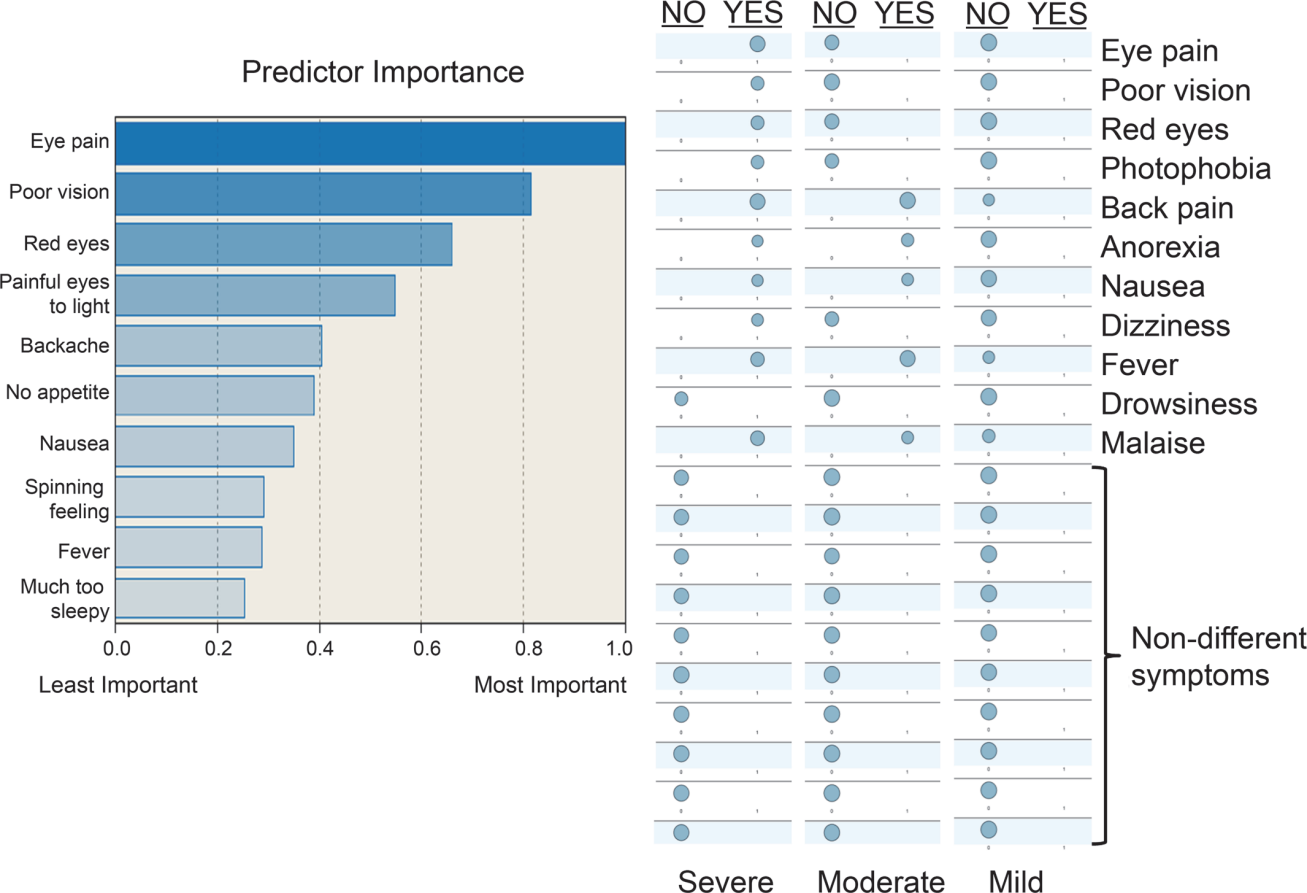
Summary of RVF symptom severity cluster analysis. With the use of two-step clustering algorithm, subjects who were seropositive for anti-RVFV IgG antibody were empirically grouped into three levels of symptom severity: mild, moderate and severe. The left panel indicates the relative importance (weight) of each symptom in assigning group membership. The right panel indicates the most typical profile of symptoms for subjects in each severity category. S2 Table provides numerical values for the symptom weights used in this classification.

In further analysis aimed at assessing the overall severity of illness, we used a statistical cluster analysis to group the 171 RVFV seropositive subjects (having recorded symptoms) according to empirically-derived symptom-complex clusters using the two-step cluster algorithm in SPSS v. 20 [63]. In the three subject clusters derived by this approach, we were able to classify subjects as having had mild RVF (N = 64, few or no symptoms reported), moderate RVF (N = 55, non-focal symptoms of acute fever), or more severe RVF (N = 52, febrile symptoms + eye symptoms +/− meningoencephalitic or hemorrhagic symptoms). In this grouping, eye symptoms emerged as the most influential in determining group assignment (Fig. 2). For purposes of analysis, members in these patient history-derived groups were categorized as: probable severe RVF (systemic + eye symptoms; Group 1), probable moderate RVF (systemic symptoms only; Group 2) and probable mild RVF (few or no symptoms; Group 3).

### Genetic and statistical analysis

Presence vs. absence of the clinical phenotype clusters plus positive RVFV serology were the phenotypes of interest. SNP genotypes were analyzed both according to a dominant model with respect to the minor allele, and according to an additive model with increasing counts of the minor allele (e.g., AA = 0, AB = 1, BB = 2); these analyses were conducted using PLINK [64] (http://pngu.mgh.harvard.edu/∼purcell/plink/). If there were fewer than 10 rare homozygotes plus heterozygotes, the Fisher’s exact test was used to define related p values for significance testing. If there were fewer than 10 homozygotes for the rare allele in either the affected or unaffected group for a given trait, the results from the additive model were not considered. In addition, the categorical cluster score trait was analyzed using Kendall’s tau to model increasing severity of the phenotype in association with increasing counts of the minor allele, using dominant and additive models as before; the analyses were conducted using SPSS version 20.

Haplotype association analyses were conducted using the proxy association method in PLINK. The most likely haplotypes were evaluated using the EM algorithm. Two statistical tests are reported: an omnibus test, which compares the distribution of most likely haplotypes in cases versus controls, and haplotype-specific tests, comparing the presence versus absence of that specific haplotype in cases versus controls. Finally, gene-level and pathway-level analyses were conducted using PLINK.

Because this was an exploratory pilot study, results significant at α = 0.10 are presented. Of note, a large number of associations (46 SNPs) were tested in our exploratory analysis, and none of the results were significant after Bonferroni correction for multiple test comparisons.

## Results

### Human subject characteristics and clinical symptom clusters

The analysis included 363 individuals, of whom 219 (67.1%) were female (Table 1). A total of 117 (32.2%) were found to be seropositive for RVFV by ELISA.

Subject- reported RVFV-associated symptoms were clustered to system-related and severity-related groupings to facilitate association analysis with genetic polymorphisms (Table 1). Many individuals (72%) reported at least one non-specific RVF-associated symptom, such as past fever (66%) or malaise (51%). Meningoencephalitis symptoms (photophobia, meningismus, vertigo/dizziness, reduced consciousness, confusion, coma, or seizures) were common and at least one symptom was reported by 44.6% of individuals. Individuals reporting at least 3 symptoms of meningoencephalitis, a more stringent classification, were much less common (8.3%). Any hemorrhagic (HE) fever-associated symptom was reported by 9.4%; three or more HE symptoms were reported by only 4 subjects (1.1%). By contrast, eye disease symptoms were commonly reported (38.6% for any single symptom; 29.5% for 2 or more symptoms).

Our computer-assisted severity group clustering, based on overall number and type of symptoms per individual, classified 28/177 (24%) seropositive subjects as having been more mildly symptomatic, 44/117 (38%) as moderately symptomatic, and 45 (38%) as more severely symptomatic (Table 1).

### Genetic associations with single polymorphisms

To analyze associations of clinical disease with individual SNPs, a single major locus model was first applied, and significance considered at α = 0.10. Using a dominant model, a number of SNPs showed association with clinical phenotypes with p < 0.10 (Table 2). We looked at several polymorphisms in complement factor H (CFH), a gene previously found to be associated with eye disease in published genome-wide association studies [65,66] as well as host susceptibility to meningococcal disease [67]. A single polymorphism in CFH (rs1061147) was associated with the presence of any eye symptom (p = 0.059). However, two other SNPs in this gene (rs1065489; rs3753396) were not significantly associated with individual symptoms or with clusters of clinical symptoms (S3 Table).

**Table 2:** Genetic association analysis results from dominant model.

Gene	SNP	Type of variant	Trait	OR	p	Fisher's exact p ^a^
CFH	rs1061147	Exon Q>P	any_eye^b^	1.337	0.05924	
IL6	rs2069849	Exon S>F	nonspecific3^c^	2.027	0.02544	
IL6R	rs4072391	3’UTR	HE_any^d^	0.4454	0.08239	0.106
IL6R	rs4072391	3’UTR	ME3^e^	0.4183	0.09002	0.126
IL6R	rs7514452	3’UTR	HE_any	0.414	0.08538	0.134
DDX58 (RIG-I)	rs2274863	Exon R>C	ME3	0.283	0.02585	
DDX58 (RIG-I)	rs2274863	Exon R>C	ME_any^f^	0.631	0.03013	
DDX58 (RIG-I)	rs2274863	Exon R>C	serology	0.637	0.05352	
MAVS	rs3746660	3’UTR	any_eye	1.452	0.04071	
MAVS	rs3746660	3’UTR	ME_any	1.406	0.05914	
MAVS	rs3746660	3’UTR	eye2^g^	1.432	0.05979	
MAVS	rs7262903	Exon R>S	serology	0.7245	0.05687	
MAVS	rs7269320	Exon—synon	serology	0.729	0.0604	
TLR7	rs864058	Exon P>L	HE_any	0.3593	0.02803	0.093
TLR7	rs864058	Exon P>L	serology	1.567	0.03197	
TLR8	rs3747414	Exon STOP	ME3	0.4688	0.06883	0.031
TLR8	rs5744088	3”UTR	serology	0.5701	0.08065	
MyD88	rs6853	3’UTR	HE_any	1.839	0.01776	
TICAM1 (TRIF)	rs2292151	Exon—synon	ME3	0.2668	0.007417	0.002
TICAM1 (TRIF)	rs2292151	Exon—synon	any^h^	0.5845	0.03989	
TICAM1 (TRIF)	rs2292151	Exon—synon	any_eye	0.686	0.05422	
TICAM1 (TRIF)	rs2292151	Exon—synon	nonspec_any^i^	0.6166	0.05474	
TICAM1 (TRIF)	rs2292151	Exon—synon	eye2	0.6696	0.062	
TICAM1 (TRIF)	rs2292151	Exon—synon	ME_any	0.7289	0.09335	

All p < 0.10 shown

^a^ Fisher’s exact test used when genotype counts were < 5

^b^ any_eye = RVFV eye disease symptoms—any

^c^ nonspec3 = RVFV non-specific—3 or more symptoms

^d^ HE-any = Hemorrhagic fever symptoms—any

^e^ ME3 = Meningoencephalitis—3 or more symptoms

^f^ ME-any = Meningoencephalitis symptoms—any

^g^ eye2 = RVFV eye disease—2 or more symptoms

^h^ any = Any symptoms

^i^ nonspec_any = RVFV non-specific symptoms

A polymorphism in the gene of the pro-inflammatory cytokine interleukin-6 (IL-6) (rs2069849) was associated with presence of 3 or more non-specific symptoms (p = 0.025, Table 2). Two SNPs in the 3’ UTR region of the IL-6 receptor were associated with meningoencephalitis or hemorrhagic symptoms with a p < 0.10 (rs4072391; rs7514452).

Also detailed in Table 2, several single polymorphisms in the RNA helicase pathway showed associations with clinical symptoms. A polymorphism in *DDX58* (RIG-I) (rs2274863) was associated with the subject report of 3 or more ME symptoms (ME3, p = 0.026), and with the past experience of any ME symptom (p = 0.03). A SNP in the 3’ UTR of the common adaptor MAVS (rs3746660) was significantly associated with the experience of any eye symptom (p = 0.041), any ME symptom (p = 0.059) and also with a history of having had two or more eye symptoms (eye2, p = 0.0598) and two different SNPs were associated with positive serology (rs7262903; rs7269320).

In the TLR pathway, TLR7 SNP rs864058 was associated with positive RVFV serology (p = 0.032) as well as a history of any hemorrhagic symptom (HE_any, p = 0.02803). TLR8 SNPs rs3747414 and rs5744088 were associated with having three or more meningoencephalitic symptoms (ME3) and positive serology, respectively. The SNP rs6853, in the adaptor molecule MyD88 which mediates signaling by both TLR7 and TLR8, showed association with the presence of at least one HE symptom (p = 0.01776). The adaptor TRIF (*TICAM1*; rs229151) was associated with ME3 (Fisher’s exact p = 0.002), as well as eye2, any eye, ME_any, and the presence of non-specific symptoms.

An additive analysis was next performed to examine the impact of multiple copies of the polymorphisms of interest. Because of the rarity of some of these phenotypes and SNP minor alleles, the dominant model (as shown in Table 2) was more significant, with a few SNPs showing robust associations in the additive model (Table 3). CCR5, RIG-I, LGP2 and IFNAR1 all had SNPs associated with clinical symptom traits at the p < 0.1 level (Table 3).

**Table 3:** Genetic association analysis results from additive model.

Gene	SNP	Type of variant	Trait	Affected	Unaffected	Chi-square statistic	p
CCR5	rs1799988	5’UTR	ME3^a^	9/9/2011	74/173/82	5.009	0.0817
DDX58 (RIG-I)	rs1133071	3’UTR	any ^b^	46/140/128	9/10/2020	5.427	0.0663
	rs1133071	3’UTR	any3 ^c^	29/96/77	26/54/71	4.901	0.0863
DHX58 (LGP2)	rs2074158	Exon R>G	serology	16/47/54	17/115/107	4.772	0.0920
IFNAR1	rs17875834	Exon R>C	any3	12/109/83	16/58/79	9.146	0.0103

All p < 0.10 shown

^a^ ME3 = Meningoencephalitis—3 or more symptoms

^b^ any = Any symptoms

^c^ any3 = Any 3 or more symptoms

### Association with phenotype clusters and severity

Next, we examined the association between the SNPs we selected for study and disease severity as determined by cluster analysis, again considering significance at α = 0.10 level. As shown in Table 4, rank correlation of SNP genotype with severity cluster scores revealed significant associations with exon SNPs in the LGP2 helicase (p = 0.08), the MAVS adaptor molecule (p = 0.047), and the interferon-receptor IFNAR1 (p = 0.013).

**Table 4:** Association results from phenotype cluster / severity analysis, using Kendall’s tau.

Gene	SNP	Type of variant	p
DHX58 (LGP2)	rs2074158	Exon R>G	0.080
MAVS	rs7262903	Exon R>S	0.047
IFNAR1	rs2257167	Exon STOP	0.013

All p < 0.10 shown

### Gene and pathway association analysis

To better understand the effect of polymorphisms in the overall genes of interest (versus specific SNP associations), we conducted gene- and pathway-level tests of association. In this analysis, all of the single SNP associations within a gene or pathway were included and significance considered at the α = 0.10 level. As shown in Table 5, TLR7 variation was associated with the presence of at least one HE symptom (p = 0.062) and TLR3 gene was associated with presence of 2 or more HE symptoms (p = 0.022). In the pathway analysis, the TLR3-TRIF pathway was associated with HE2 (p = 0.035) and ME3 (p = 0.0176), the TLR7-MyD88 pathway and TLR8-MyD88 pathways were both associated with HE_any (p = 0.07; p = 0.039) and the combined TLR7-TLR8-MyD88 pathway was associated with presence of at least one HE symptom (p = 0.0458). The IL6-IL6R pathway was associated with presence of 3 or more non-specific symptoms (p = 0.065).

**Table 5:** Gene and pathway—level associations.

Gene / Pathway	Trait	p
*Single genes*		
TLR7	HE_any ^a^	0.06194
TLR3	HE2 ^b^	0.02198
*Pathways*		
TLR3-TRIF	HE2	0.0348
TLR7-TLR8-MyD88	HE_any	0.0458
TLR3-TRIF	ME3 ^c^	0.0176
TLR7-MyD88	HE_any	0.0701
TLR8-MyD88	HE_any	0.0394
IL6-IL6R	Nonspec3 ^d^	0.06499

All p < 0.10 shown

^a^ HE-any = Hemorrhagic fever symptoms—any

^b^ HE2 = Hemorrhagic fever—2 or more symptoms

^c^ ME3 = Meningoencephalitis—3 or more symptoms

^d^ nonspec3 = RVFV non-specific—3 or more symptoms

### Haplotype analysis

Finally, we conducted a haplotype analysis based on the most likely haplotype phases using the EM algorithm as implemented by the proxy association method in PLINK, considering significance at α = 0.10. First, we found that haplotypes in TLR3 had a different distribution in individuals with and without HE2 (Table 6); the overall distribution was significantly different (p = 0.0102). The TG haplotype was associated with a 5-fold increased risk of HE2 (p = 0.00775), and the AG haplotype had a significant protective effect (p = 0.0103). These results confirm the gene-level association between HE2 and TLR3 in Table 5. Second, we found haplotypes in MAVS were distributed differently in individuals with and without Any3 (omnibus p = 0.0514), with the GCT haplotype resulting in a 1.88 increased risk of Any3 (p = 0.00295). Though single SNP analyses of MAVS revealed associations with other clinical phenotypes, there were no associations observed with Any3, suggesting that an untyped polymorphism on the GCT haplotype may be associated with Any3.

**Table 6:** Haplotype association analysis.

*Trait: HE2* ^a^ *Gene: TLR3, SNPs: rs5743310,rs3775296, Omnibus haplotype test statistic **p = 0.0102***			
**Haplotype**	**Overall frequency**	**OR**	**p**
AT	0.0227	2.00	0.315
TG	0.105	5.72	**0.00775**
AG	0.668	0.161	**0.0103**
*Trait: Any3* ^b^ *Gene: MAVS, SNPs: rs17857295,rs7269320,rs3746660, Omnibus haplotype test statistic **p = 0.0514***			
**Haplotype**	**Overall frequency**	**OR**	**p**
GCT	0.0894	1.88	**0.00295**
CCT	0.135	1.11	0.209
GTC	0.109	0.844	0.465
CTC	0.268	1.109	0.742
GCC	0.462	1.67	0.105
CCC	0.348	0.707	0.222

^a^ HE2 = Hemorrhagic fever—2 or more symptoms

^b^ any3 = Any 3 or more symptoms

## Discussion

In this study we examined polymorphisms in human genes of the innate immune system using diverse approaches and demonstrated association with a variety of clinical phenotypes in an ethnically Somali population of long-term residents in a RVFV endemic area. Our analysis included documentation of symptom recall using a structured interview administered by trained study personnel and we acknowledge that there may be inaccuracies with self-reported symptoms including memory lapses, selective recall of more severe symptomatology and other potential biases which are difficult to control in a retrospective study of this nature. Additional epidemiological factors associated with seropositivity and with severity of disease in this population are described elsewhere [11]. We analyzed a total of 46 SNPs in 16 genes (*CFH, IL6, IL6R, IFIH1, DDX58, DHX58, MAVS, CCR5, TLR3, TLR7, TLR8, MYD88, TICAM1, IFNB, IFNAR1 CD209*), and have identified innate immunity pathways that may play an important role in the pathogenesis of clinical RVF associated symptoms. These genes included those for the RNA helicases RIG-I (*DDX58*), LGP2 (*DHX58*) and their common adaptor MAVS (also called IPS-1) as well as endosomal Toll-like receptors TLR3, TLR7 and TLR8 and their signaling adaptors MyD88 and TRIF.

A strong association was observed in our analysis of the inflammatory cytokine IL-6 and its receptor, IL6R. We found association of the IL6 SNP and 3 of the 4 IL6R SNPs in our single gene analysis (Table 2). In an analysis of all SNPs in the *IL6* and *IL6R* pathway, a significant association was found with non—specific symptoms including fever, anorexia, and backache. Therefore, although our data does not show a strong association between IL-6 and severe RVF symptoms, there is likely a role for IL-6 response, along with those for other inflammatory cytokines, in the pathogenesis of severe RVF. We have previously shown that IL-6 is one of several inflammatory responses to RVFV infection in a murine model of mucosal RVFV infection [50]. It is possible that robust IL-6 responses may lead to a cytokine “storm” via IL-6 receptor signaling, resulting in more severe clinical pathology such as hepatic inflammation, encephalitis, and risk for death.

One gene that was of interest, based on previously published GWAS studies was serum complement factor H. Several studies have shown an association of CFH mutations with age related macular degeneration [68–70] as well as with other eye diseases including uveitis [71]. Retinitis is a serious long-term complication of human RVF and we have previously observed prevalence as high as 21% in our study population [10,59]; therefore, we hypothesized that CFH may contribute to the pathogenesis of retinal disease. Surprisingly, we did not see a strong association of any of the 6 SNPs in the CFH gene with RVF specific eye disease symptoms, although one SNP (rs1061147) showed weak association with a cluster of general eye symptoms (Table 2).

As other viruses, including a related member of the *Phlebovirus* group, the ssRNA virus Punta Toro virus (PTV), have been associated with TLR activation [48,72], we decided to look for associations of clinical RVF symptoms with common polymorphisms in TLRs and signaling adaptor molecule genes. Although we did not find an association with individual SNPs in TLR3, we did find a TLR3 gene-level as well as haplotype association with having had two or more symptoms of hemorrhagic fever. Also, there was a pathway association between TLR3-TRIF SNPs and multiple symptoms of hemorrhagic fever, as well as 3 or more symptoms of meningoencephalitis, which suggests that this innate pathway is important in the pathogenesis of RVFV-associated severe disease. Although these association results were not all significant at α = 0.05, the consistency of association across phenotypes suggests the associations are robust and indicate a role in RVFV pathology. This was not surprising as there has been a clear association between TLR3 mediated innate responses and poor outcomes in a murine model of PTV [48]; however, in a murine model of mucosal RVFV infection we did not see an impact of the TLR3/TRIF pathway on severe disease or type I IFN responses [50]. A recent paper found Toll-7 dependence of RVFV induced autophagy in Drosophila and MyD88 dependence in a human osteosarcoma cell line [73], although the role of these pathways in human primary immune cell autophagy or immune responses remains unclear. In previous studies we also did not see TLR7 or TLR8 dependence for IFN and other cytokine responses to RVFV, yet in this genetic analysis we do see associations between polymorphisms in human TLR7 and TLR8 with clinical symptoms at the level of individual SNPs, as well as single gene and pathway analysis [50]. We conclude that the impact of the endosomal TLRs, including TLR3, TLR7 and TLR8 in the innate responses to RVFV and the pathogenesis of severe RVF is unclear. There may be differences between the utilization of endosomal TLRs for viral sensing at the cellular level and the impact of these important innate receptors at the whole organism level. Also, it is increasingly being recognized that there are important differences between mouse and human innate receptor activity in health and disease which may be contributing to the differences that we observe between laboratory studies and analysis of human field collected samples.

We have previously shown the importance of the RNA helicases RIG-I and MDA5 as well as the common signaling adaptor MAVS (also known as IPS-1) in RVFV induced IFN responses [50]. In a single gene analysis, we observed a trend towards a significant impact of one individual RIG-I (*DDX58*) SNP located in the exon of the gene. In the additive model, significant correlations were found between the helicase family member LGP2 (*DHX58*) and serology and between a 3’UTR SNP in RIG-I and any symptom. Interestingly, in a symptom cluster analysis both LGP2 (*DHX58*) and MAVS showed association and MAVS also showed association using a haplotype an analysis. These findings point out the challenge of single allelic association testing; whereas using more complex haplotype and cluster analysis demonstrated association of this well established viral innate sensing pathway with clinical phenotypes in RVF.

The type I interferon receptor, IFNAR, is formed by class II helical cytokine receptor family members IFNAR1 and IFNAR2 [74–76]. Although the role of type I IFN in host defense to multiple viruses is well established, and the role of IFNAR in modulating susceptibility and severity of disease has been established in multiple models of viral infection, including RVFV [77], no previous human studies have shown a correlation of genetic polymorphisms in the IFNAR genes with disease phenotype in RVF. Other groups have shown association with polymorphisms in IFNAR1 and Hepatitis B and C infection and disease [78–80]. Our current studies found significant associations between two polymorphisms (rs2257167, rs17875834) and disease phenotypes using an additive model and phenotype cluster / severity analysis.

Our findings point to important innate immune pathways in the pathogenesis of RVF associated symptoms. Polymorphisms in TLR3, TLR7, TLR8, MyD88, TRIF, MAVS, and RIG-I were repeatedly associated with severe symptomatology, suggesting that these genes may have a robust association with RVFV-associated clinical outcomes. Future studies to further explore the importance of these pathways in RVFV associated disease in different populations as well as correlation with in vivo and in vitro models of RVF are warranted.

## Supporting Information

S1 Checklist STROBE Checklist. (DOCX)

S1 Table Allele frequencies for SNPs included in analysis. (DOCX)

S2 Table Display of weighting factors used for symptom severity score classification for Groups 1–3 used in the SNP association analysis. (DOCX)

S3 Table Additional genetic association analysis results from dominant model. (DOCX)
