# Supplementary material for: Association of Symptoms and Severity of Rift Valley Fever with Genetic Polymorphisms in Human Innate Immune Pathways
Source: PLoS Negl Trop Dis. 2015 Mar 10;9(3):e0003584. doi: 10.1371/journal.pntd.0003584 (PMC4355584; doi:10.1371/journal.pntd.0003584)
Supplement: S1 Table — (DOCX) [file pntd.0003584.s002.docx]

**S1 Table. Allele frequencies for SNPs included in analysis.**

| **CHR**^1^ | **Gene** | **SNP** | **allele 1** | **allele 2** | **MAF**^2^ |
| --- | --- | --- | --- | --- | --- |
| 1 | CFH | rs1061147 | A | C | 0.4588 |
| 1 | CFH | rs1065489 | T | G | 0.0948 |
| 1 | CFH | rs3753396 | G | A | 0.1009 |
| 1 | CFH | rs488738 | T | A | 0.2192 |
| 1 | CFH | rs515299 | A | C | 0.2048 |
| 1 | CFH | rs534399 | A | C | 0.2237 |
| 1 | IL6R | rs2229238 | T | C | 0.1204 |
| 1 | IL6R | rs4072391 | T | C | 0.1416 |
| 1 | IL6R | rs4379670 | T | A | 0.1187 |
| 1 | IL6R | rs7514452 | C | T | 0.1210 |
| 2 | IFIH1 | rs10930046 | C | T | 0.1640 |
| 2 | IFIH1 | rs1990760 | T | C | 0.2185 |
| 2 | IFIH1 | rs3747517 | G | A | 0.4829 |
| 3 | CCR5 | rs1799988 | T | C | 0.4874 |
| 3 | MYD88 | rs6853 | G | A | 0.3494 |
| 4 | TLR3 | rs3775296 | T | G | 0.2272 |
| 4 | TLR3 | rs5743310 | T | A | 0.1050 |
| 7 | IL6 | rs2069849 | T | C | 0.0588 |
| 9 | DDX58 | rs1133071 | C | T | 0.3643 |
| 9 | DDX58 | rs12006123 | A | G | 0.1256 |
| 9 | DDX58 | rs2274863 | C | T | 0.1521 |
| 9 | DDX58 | rs3205166 | C | A | 0.4315 |
| 9 | DDX58 | rs3739674 | C | G | 0.4060 |
| 9 | IFNB1 | rs1051922 | T | C | 0.4121 |
| 17 | DHX58 | rs2074158 | A | G | 0.3299 |
| 19 | CD209 | rs4804803 | G | A | 0.3380 |
| 19 | TICAM1 | rs2292151 | T | C | 0.2095 |
| 20 | MAVS | rs17857295 | G | C | 0.2454 |
| 20 | MAVS | rs3746660 | T | C | 0.2283 |
| 20 | MAVS | rs7262903 | A | C | 0.3642 |
| 20 | MAVS | rs7269320 | T | C | 0.3810 |
| 21 | IFNAR1 | rs17875834 | T | C | 0.3135 |
| 21 | IFNAR1 | rs17875863 | A | G | 0.2215 |
| 21 | IFNAR1 | rs2257167 | C | G | 0.2694 |
| 21 | IFNAR1 | rs2834202 | G | A | 0.1313 |
| X | TLR7 | rs5741881 | G | A | 0.0422 |
| X | TLR7 | rs864058 | T | C | 0.2457 |
| X | TLR8 | rs2159377 | T | C | 0.1566 |
| X | TLR8 | rs3747414 | A | C | 0.2938 |
| X | TLR8 | rs3764880 | G | A | 0.3653 |
| X | TLR8 | rs5744077 | G | A | 0.1569 |
| X | TLR8 | rs5744080 | T | C | 0.4191 |
| X | TLR8 | rs5744081 | A | C | 0.0140 |
| X | TLR8 | rs5744084 | A | C | 0.1529 |
| X | TLR8 | rs5744088 | C | G | 0.1190 |
| X | TLR8 | rs5979764 | C | G | 0.0225 |

^1^CHR = chromosome number, ^2^MAF = minor allele frequency (frequency of less common allele)
