# Supplementary material for: Association of Symptoms and Severity of Rift Valley Fever with Genetic Polymorphisms in Human Innate Immune Pathways
Source: PLoS Negl Trop Dis. 2015 Mar 10;9(3):e0003584. doi: 10.1371/journal.pntd.0003584 (PMC4355584; doi:10.1371/journal.pntd.0003584)

**S2 Table.** Display of weighting factors used for symptom severity score classification for Groups 1 - 3 used in the SNP association analysis**.**


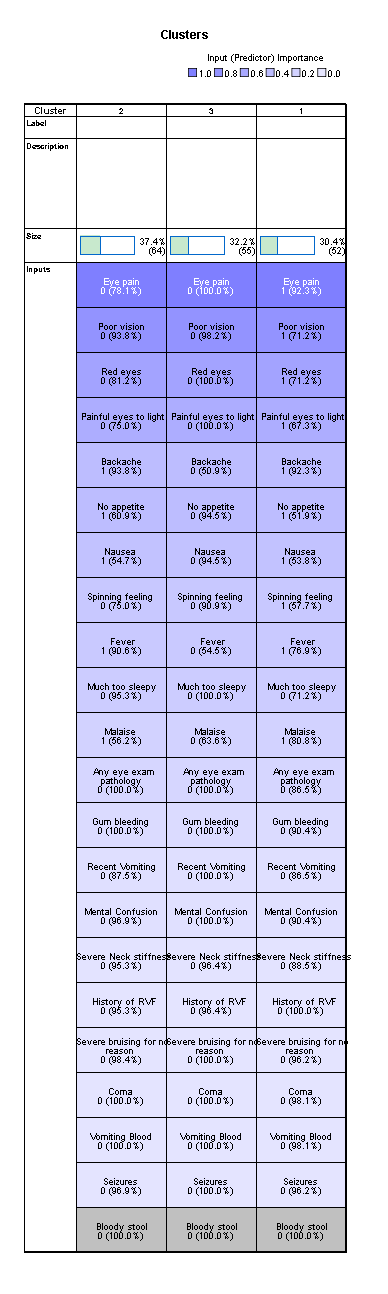

Supplement: S2 Table — (DOCX) [file pntd.0003584.s003.docx]
