# Supplementary material for: Association of Symptoms and Severity of Rift Valley Fever with Genetic Polymorphisms in Human Innate Immune Pathways
Source: PLoS Negl Trop Dis. 2015 Mar 10;9(3):e0003584. doi: 10.1371/journal.pntd.0003584 (PMC4355584; doi:10.1371/journal.pntd.0003584)
Supplement: S3 Table — (DOCX) [file pntd.0003584.s004.docx]

**S3 Table. Additional genetic association analysis results from dominant model.**

| **Gene** | **SNP** | **Type of variant** | **Trait** | **OR** | **p** |
| --- | --- | --- | --- | --- | --- |
| CFH | rs1065489 | Exon G>A | any ^a^ | 0.920 | 0.8342 |
|  |  |  | any eye ^b^ | 0.932 | 0.7895 |
|  |  |  | eye2 ^c^ | 1.013 | 0.9637 |
|  |  |  | HE-any ^d^ | 1.331 | 0.4737 |
|  |  |  | ME_any ^e^ | 0.706 | 0.1856 |
|  |  |  | ME3 ^f^ | 1.368 | 0.4595 |
|  |  |  | nonspec_any ^g^ | 0.801 | 0.5413 |
|  |  |  | nonspecific3 ^h^ | 1.190 | 0.4985 |
| CFH | rs3753396 | Exon - synon | any | 0.962 | 0.9222 |
|  |  |  | any eye | 1.093 | 0.7281 |
|  |  |  | eye2 | 1.151 | 0.5995 |
|  |  |  | HE-any | 1.234 | 0.5979 |
|  |  |  | ME_any | 0.825 | 0.4479 |
|  |  |  | ME3 | 1.27 | 0.5726 |
|  |  |  | nonspec_any | 0.842 | 0.6346 |
|  |  |  | nonspecific3 | 1.119 | 0.6546 |
|  |  |  | serology | 0.879 | 0.6389 |

^a^ any = Any symptoms

^b^ any_eye = RVFV eye disease symptoms – any

^c^ eye2 = RVFV eye disease – 2 or more symptoms

^d^ HE-any = Hemorrhagic fever symptoms – any

^e^ ME-any = Meningoencephalitis symptoms – any

^f^ ME3 = Meningoencephalitis – 3 or more symptoms

^g^ nonspec_any = RVFV non-specific symptoms

^h^ nonspec3 = RVFV non-specific – 3 or more symptoms
